# Supplementary material for: Mitochondrial damage triggers the concerted degradation of negative regulators of neuronal autophagy
Source: Nat Commun. 2025 Aug 9;16:7367. doi: 10.1038/s41467-025-62379-5 (PMC12335601; doi:10.1038/s41467-025-62379-5)
Supplement: Supplementary file 1 — Supplementary Information [file 41467_2025_62379_MOESM1_ESM.pdf]

Mitochondrial damage triggers the concerted degradation of negative regulators of neuronal autophagy

Bishal Basak and Erika L.F. Holzbaur

SUPPLEMENTARY INFORMATION

Supplemental S1

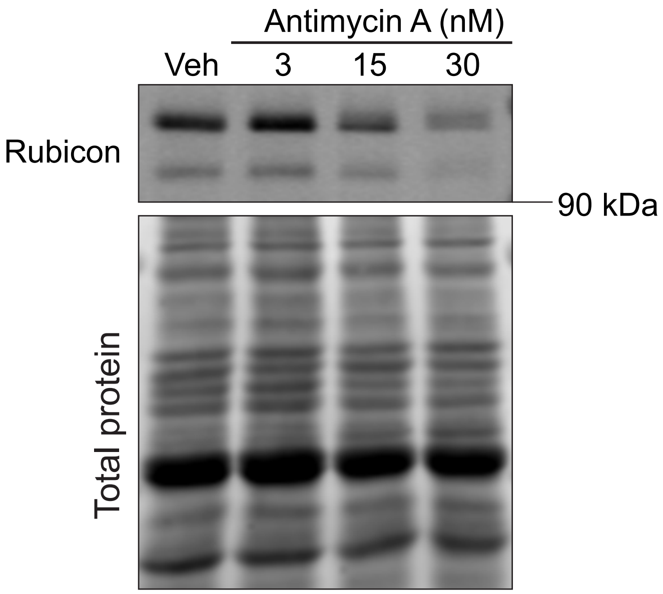

Supplemental Figure S1:
Mitochondrial damage induces parallel degradation of both the putative isoforms of Rubicon. Representative western blot from lysates of WT cortical neurons treated with vehicle (EtOH) or with increasing concentrations of Ant A (3 nM, 15 nM or 30 nM) for 2 hrs and probed for Rubicon. A doublet is seen on the immunoblot.

Supplemental S2

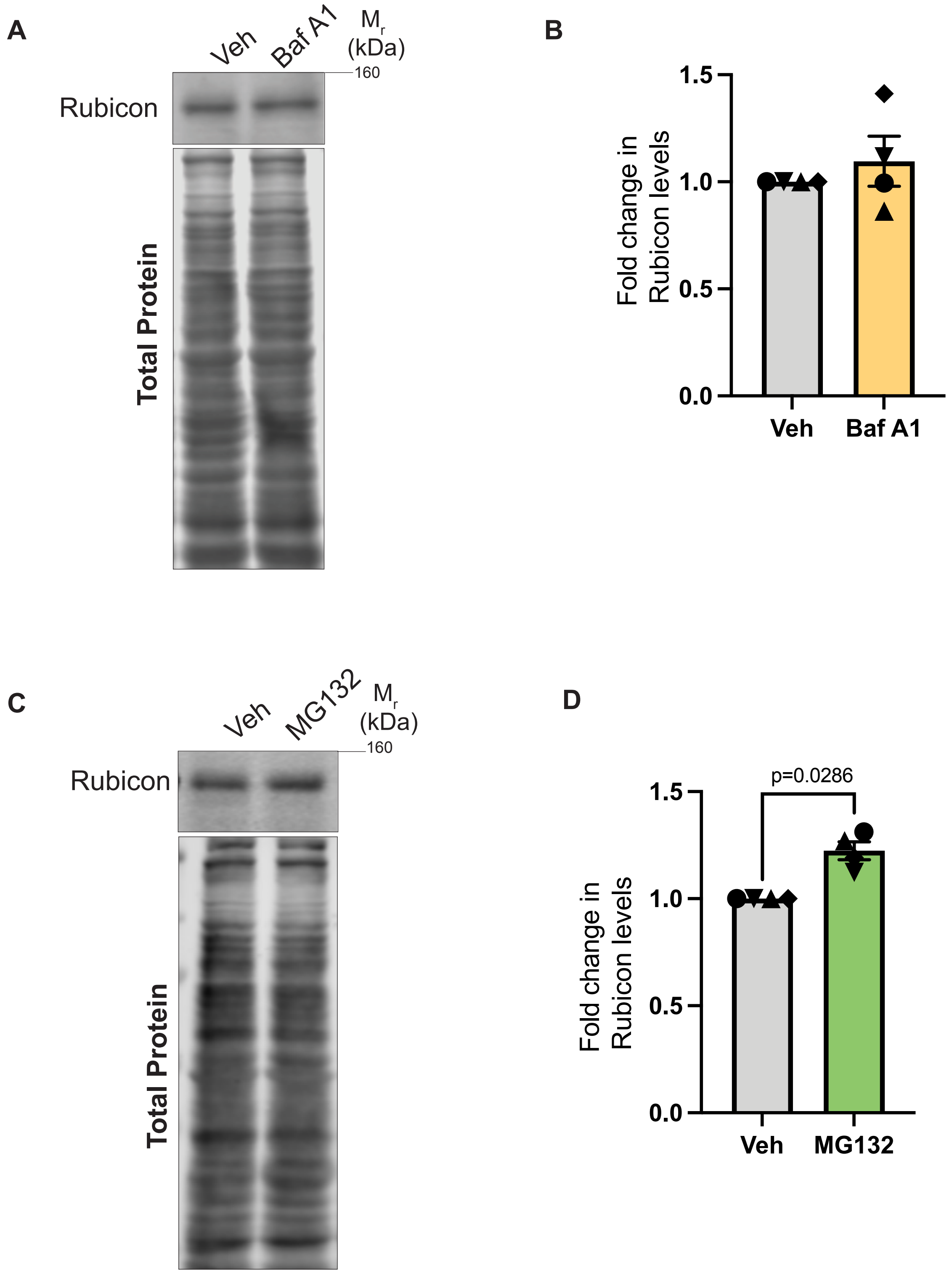

Supplemental Figure S2:

(A, B) Rubicon levels are unaltered upon blocking basal autophagy in HeLa cells. (A) Representative western blot from lysates of HeLa cells treated with vehicle (DMSO) or 500 nM Baf A1 for 3 hrs. (B) Rubicon band intensity normalized to total protein intensity from lysates of HeLa cells treated with DMSO or Baf A1 for 3 hrs. (C, D) Rubicon levels are not regulated by the proteasome in HeLa cells under basal conditions. (C) Representative western blot from lysates of HeLa cells treated with vehicle (EtOH) or 10 μM MG132 for 3 hrs. (D) Rubicon band intensity normalized to total protein intensity from lysates of HeLa cells treated with EtOH or MG132 for 3 hrs. All panels: N=4 experiments, Mann-Whitney test

Error bars indicate S.E.M.

Supplemental S3

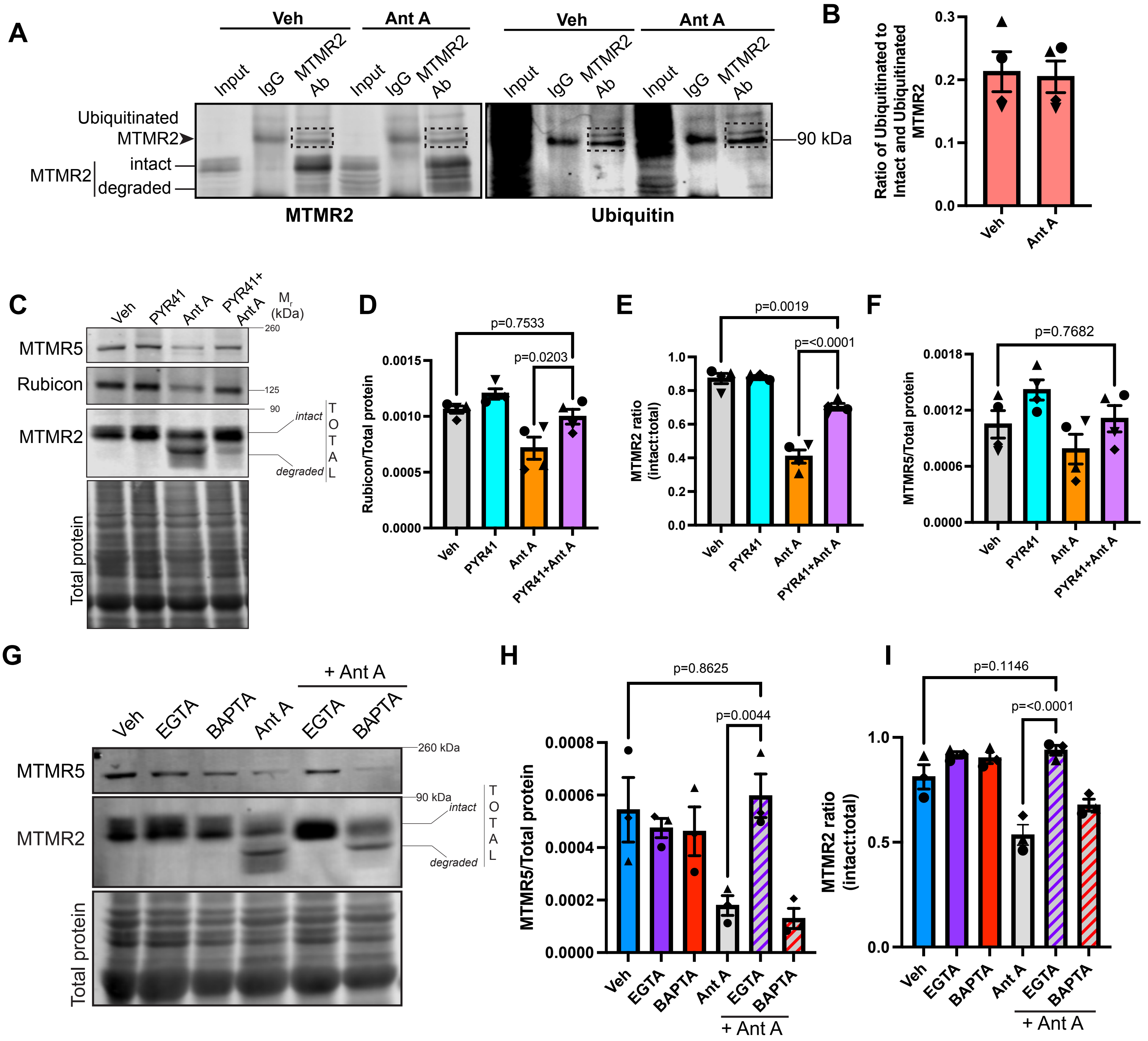

Supplemental Figure S3:

(A-B) Ubiquitination of MTMR2 in neurons. (A) Representative western blot from wild type cortical neuronal lysates treated with vehicle (EtOH) or 30 nM Ant A for 2 hrs and immunoprecipitated with MTMR2 antibody or control mouse IgG. (B) Quantification of the ratio of ubiquitinated to intact and ubiquitinated MTMR2 upon immunoprecipitation of MTMR2 in neurons treated with EtOH or Ant A (N=4 experiments, two-tailed unpaired t-test). (C-F) Blocking Ub-E1 activity by PYR41 suppresses the degradation of MTMR5/2 and Rubicon during mitochondrial damage. (C) Representative western blots from lysates of WT murine embryonic cortical neurons treated with vehicle (DMSO) or 10 μM PYR41 for 1 hr followed by an additional treatment with vehicle (EtOH) or 30 nM Ant A for 2 hrs. (D) Rubicon band intensity normalized to total protein from WT neurons treated with DMSO/PYR41 and EtOH/Ant A. (E) Ratio of intact MTMR2 normalized to total (intact + degraded) band intensities from WT neurons treated with DMSO/PYR41 and EtOH/Ant A. (F) MTMR5 band intensity normalized to total protein from WT neurons treated with DMSO/PYR41 and EtOH/Ant A (C-F: N=4 experiments, One way ANOVA with Sidak's multiple comparison test). (G-I) Blocking Ca^{2+} dependent proteolysis completely rescues the degradation of MTMR5 and MTMR2. (G) Representative western blot from lysates of WT embryonic cortical neurons treated with vehicle (water, DMSO) or 5 mM EGTA or 20 μM BAPTA for 1 hr followed by an additional treatment with vehicle (EtOH) or 30 nM Ant A for 2 hrs. (H) MTMR5 band intensity normalized to total protein from WT neurons treated with vehicle/ EGTA/ BAPTA and EtOH/Ant A. (I) Ratio of intact MTMR2 normalized to total (intact + degraded) band intensities from WT neurons treated with vehicle/EGTA/ BAPTA and EtOH/Ant A (G-I: N=3 experiments, One way ANOVA with Sidak's multiple comparison test).
Error bars indicate S.E.M.

Supplemental S4

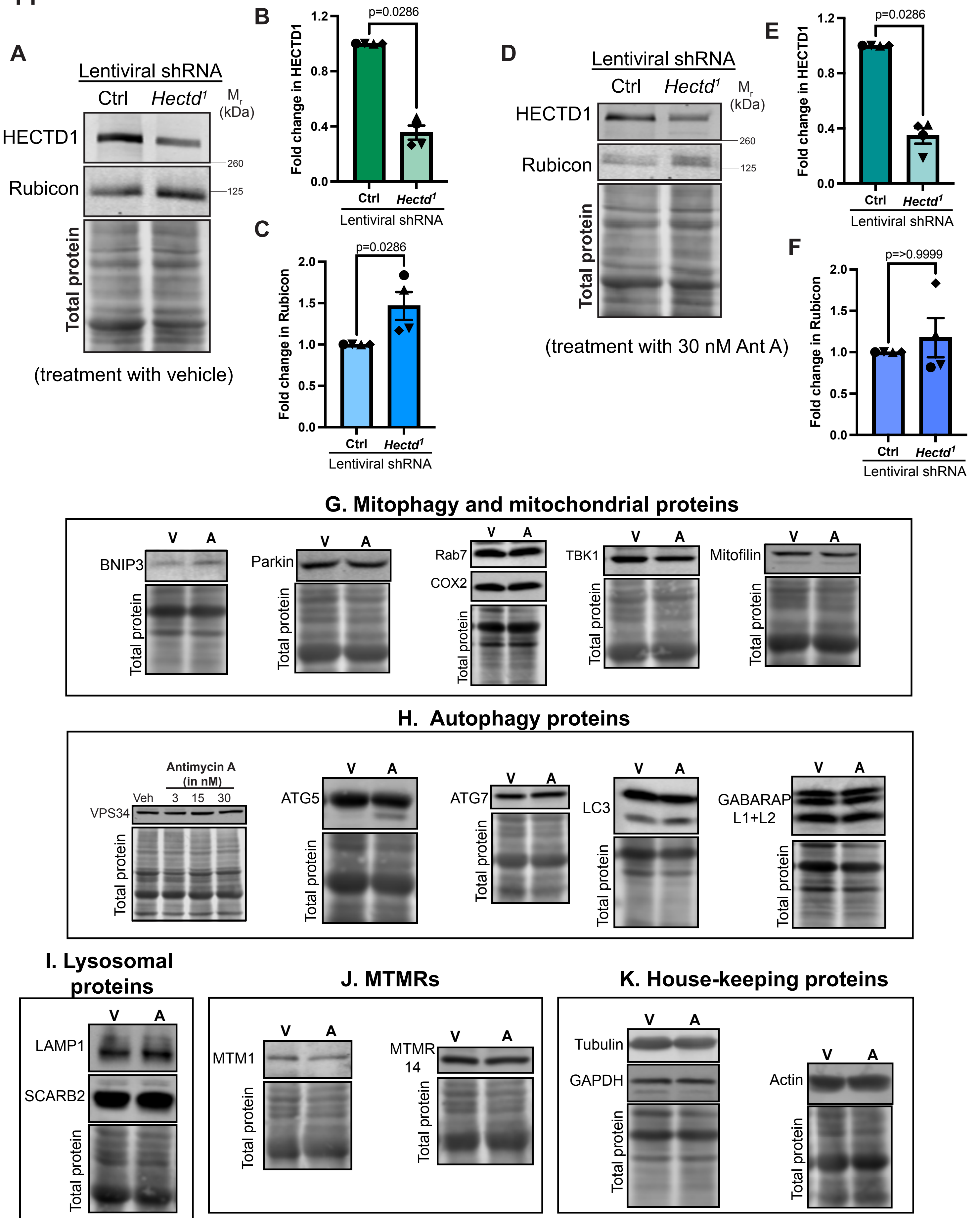

[For **G-K**: V represents vehicle, A represents 30 nM Antimycin A]

Supplemental Figure S4:

(A-C): HECTD1 ubiquitinates Rubicon under basal conditions. (A) Representative western blot from lysates of wild type cortical neurons transduced with lentivirus containing shRNA against HECTD1 or non-targeting control, and treated with vehicle (EtOH) for 2 hrs. (B) Fold change in HECTD1 levels in neurons transduced with lentivirus containing shRNA against HECTD1 or non-targeting control. (C) Fold change in Rubicon levels in neurons transduced with lentivirus containing shRNA against HECTD1 or non-targeting control. (D-F): Upon mitochondrial damage HECTD1 does not ubiquitinate Rubicon in neurons. (D) Representative western blot from lysates of wild type cortical neurons transduced with lentivirus containing shRNA against HECTD1 or non-targeting control, and treated with 30 nM Ant A for 2 hrs. (E) Fold change in HECTD1 levels in neurons transduced with lentivirus containing shRNA against HECTD1 or non-targeting control, and treated with Ant A. (F) Fold change in Rubicon levels in neurons transduced with lentivirus containing shRNA against HECTD1 or non-targeting control, and treated with Ant A. (A-F: N=4 experiments, Mann-Whitney test. Error bars indicate S.E.M.)

(G) Representative western blots from lysates of WT murine embryonic cortical neurons treated with EtOH or Ant A for 2 hrs, and probed for mitophagy and mitochondrial proteins. (H) Representative western blots from lysates of WT murine embryonic cortical neurons treated with EtOH or Ant A for 2 hrs, and probed for autophagy proteins. (I) Representative western blot from lysates of WT murine embryonic cortical neurons treated with EtOH or Ant A for 2 hrs, and probed for lysosomal proteins. (J) Representative western blots from lysates of WT murine embryonic cortical neurons treated with EtOH or Ant A for 2 hrs, and probed for MTMRs. (K) Representative western blots from lysates of WT murine embryonic cortical neurons treated with EtOH or Ant A for 2 hrs, and probed for house-keeping proteins. (For G-K: experiments were performed thrice, and representative images from one replicate is shown here).

Supplemental S5

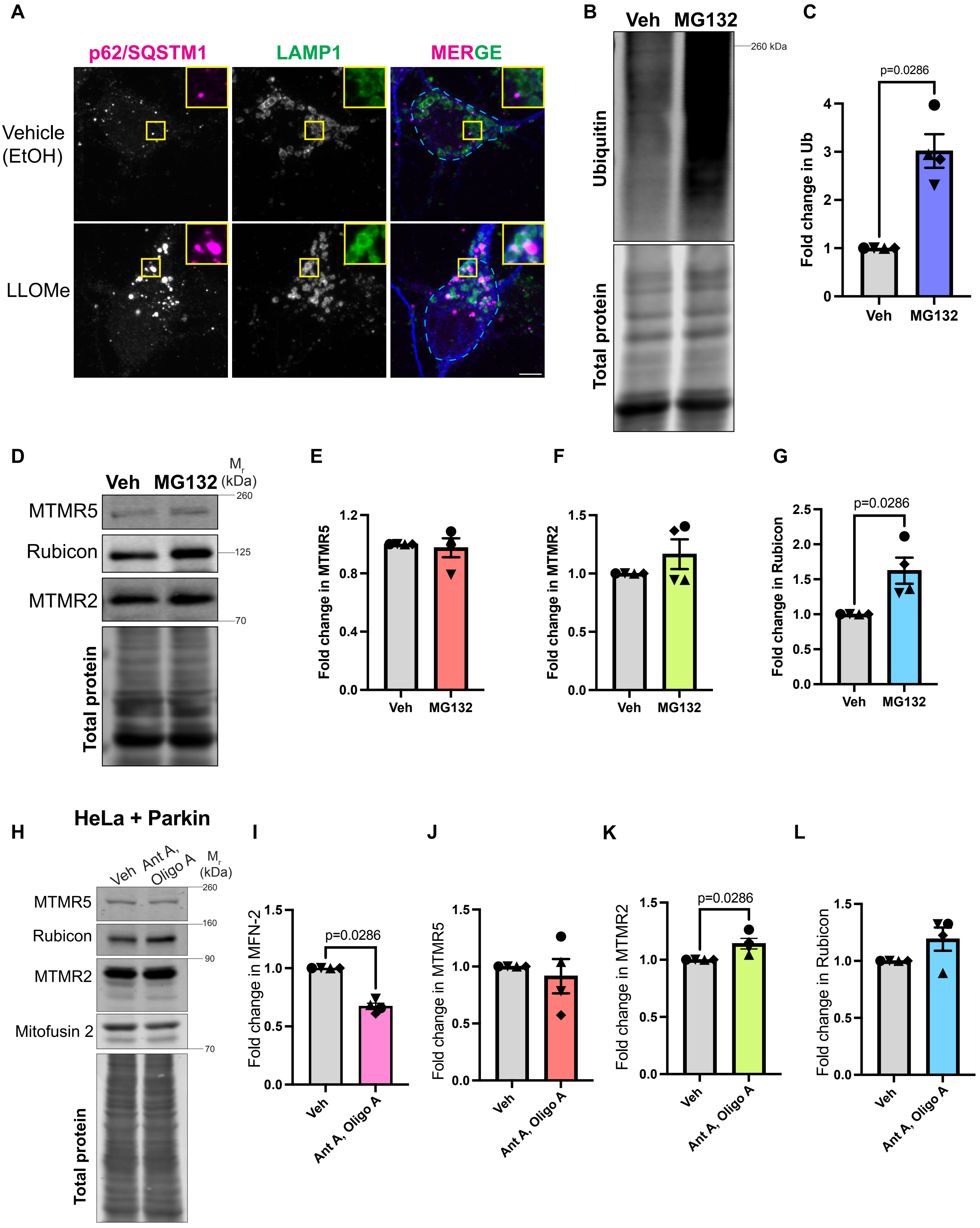

Supplemental Figure S5:
(A) Representative max projection of neurons treated with vehicle (EtOH) or 1 mM LLoMe for 2 hrs, fixed and immunostained for p62 (magenta), LAMP1 (green) and the neuronal marker MAP2 (blue). Outline of the soma is marked. Yellow boxes indicate inset regions. Scale bar= 5 µm. (The experiment was repeated thrice, and representative images from one biological replicate are shown.) (B- G) Proteotoxic stress does not induce the degradation of MTMR5, MTMR2 and Rubicon. (B) Representative western blots from lysates of WT murine embryonic cortical neurons treated with either Veh (vehicle- EtOH) or 10 µM MG132 for 3 hours, and probed for ubiquitin (Ub) (C) Fold change in Ub levels upon treatment of neurons with 10 µM MG132 as compared to with vehicle. (D) Representative western blots from lysates of WT murine embryonic cortical neurons treated with either Veh (vehicle- EtOH) or 10 µM MG132 for 3 hours. (E) Fold change in MTMR5 levels upon treatment of neurons with MG132 as compared to with vehicle. (F) Fold change in MTMR2 levels upon treatment of neurons with MG132 as compared to with vehicle. (G) Fold change in Rubicon levels upon treatment of neurons with MG132 as compared to with vehicle. (B-G: N=4 experiments, Mann-Whitney test). (H-L): HeLa cells, transfected with Parkin, activate Pink1/Parkin-dependent mitophagy in response to mitochondrial damage but not MitoSR. (H) Representative western blot from lysates of HeLa cells transfected with an untagged construct of human Parkin and then treated with 10 µM Ant A, 10 µM Oligo A or vehicle (EtOH, DMSO) for 5 hrs. (I) Fold change in Mitofusin-2 levels upon treatment of Parkin-transfected HeLa cells with 10 µM Ant A, 10 µM Oligo A as compared to with vehicle. (J) Fold change in MTMR5 levels upon treatment of Parkin-transfected HeLa cells with 10 µM Ant A, 10 µM Oligo A as compared to with vehicle. (K) Fold change in MTMR2 levels upon treatment of Parkin-transfected HeLa cells with 10 µM Ant A, 10 µM Oligo A as compared to with vehicle. (L) Fold change in Rubicon levels upon treatment of Parkin-transfected HeLa cells with 10 µM Ant A, 10 µM Oligo A as compared to with vehicle. (H-L: N=4 experiments, Mann-Whitney test)
Error bars indicate S.E.M.

Supplemental S6

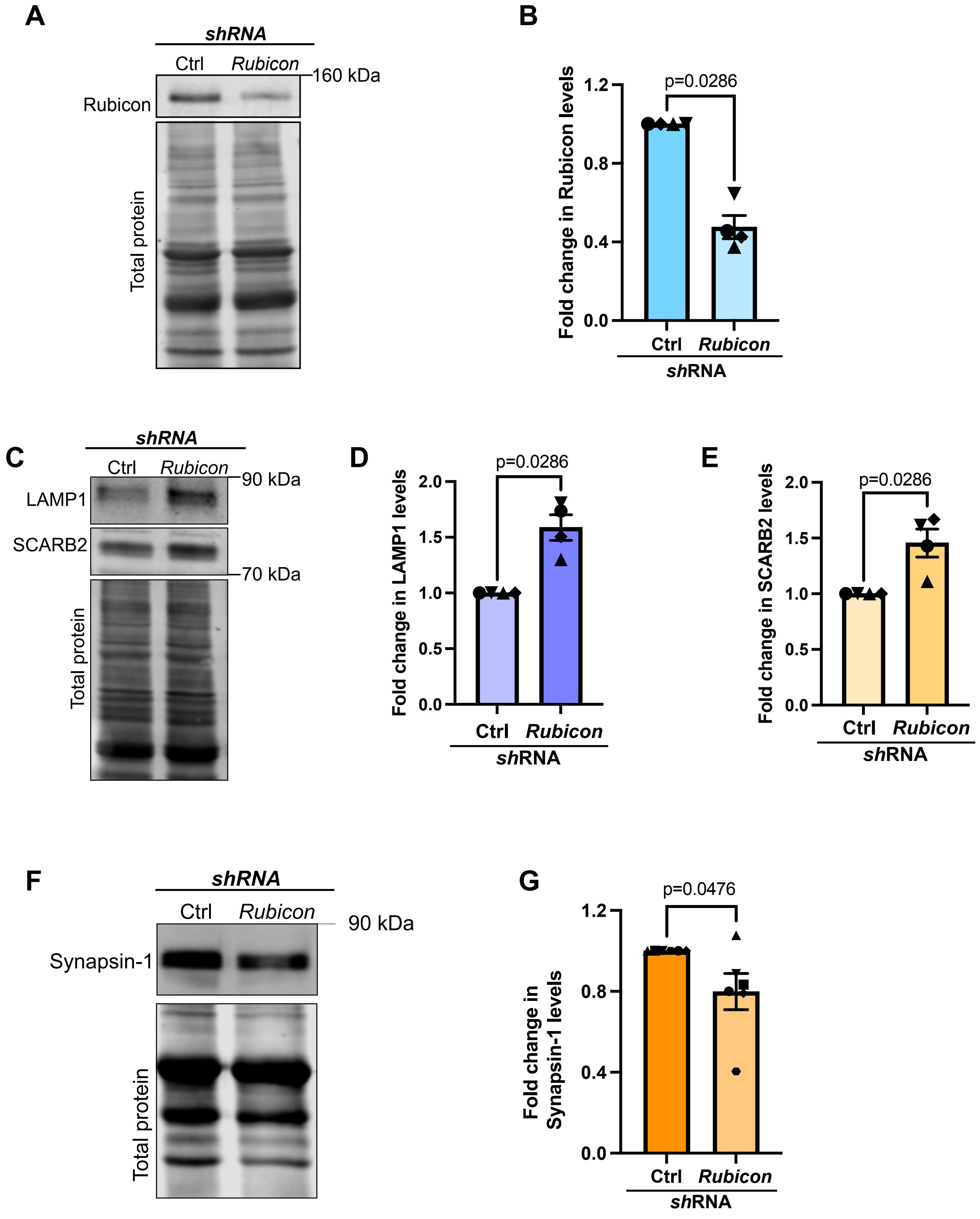

Supplemental Figure S6:
(A-B): Knockdown of Rubicon in WT cortical neurons to determine its role in regulating basal autophagy and mitophagy. (A) Representative western blot from lysates of WT cortical neurons nucleofected with control or *Rubicon* shRNA plasmid, and probed for Rubicon. (B) Fold change in Rubicon levels in neurons nucleofected with control or *Rubicon* shRNA plasmid (N= 4 experiments, Mann-Whitney test) (C-E): Rubicon negatively influences lysosomal biogenesis in neurons. (C) Representative western blot from lysates of WT cortical neurons nucleofected with control or *Rubicon* shRNA plasmid, and probed for LAMP1 and SCARB2. (D) Fold change in LAMP1 levels in neurons nucleofected with control or *Rubicon* shRNA plasmid. (E) Fold change in SCARB2 levels in neurons nucleofected with control or *Rubicon* shRNA plasmid. (C-E: N= 4 experiments, Mann-Whitney test) (F-G): Depletion of Rubicon promotes autophagic turnover of Synapsin-1. (F) Representative western blot from lysates of WT cortical neurons nucleofected with control or *Rubicon* shRNA plasmid, and probed for Synapsin-1. (G) Fold change in Synapsin-1 levels in neurons nucleofected with control or *Rubicon* shRNA plasmid (N= 6 experiments, Mann-Whitney test)
Error bars indicate S.E.M.

Supplemental S7

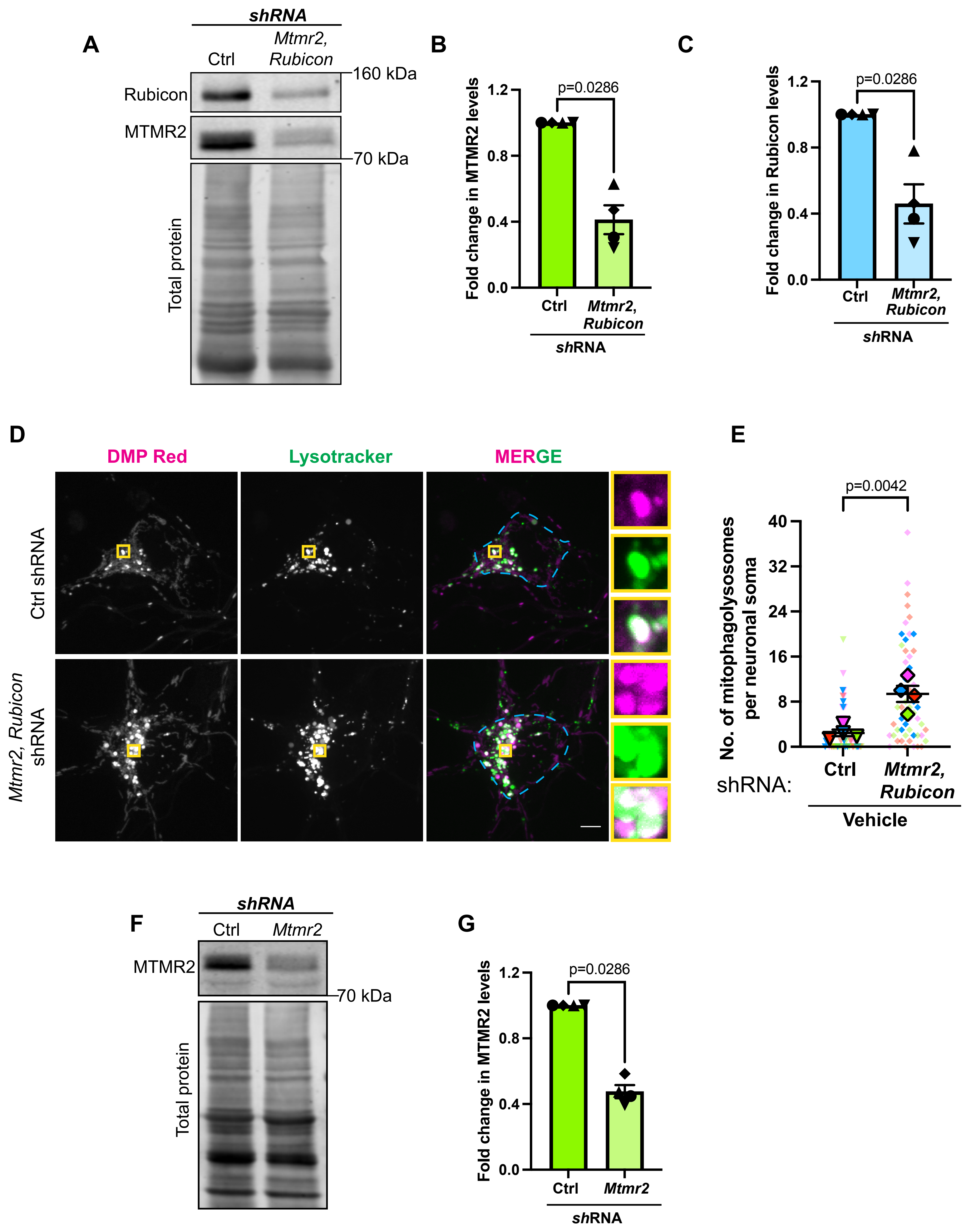

Supplemental Figure S7:
(A-C) Knockdown of MTMR2 and Rubicon in WT cortical neurons to determine their role in regulating mitophagy. (A) Representative western blot from lysates of WT cortical neurons nucleofected with control or *Mtmr2* and *Rubicon* shRNA plasmids, and probed for MTMR2 and Rubicon. (B) Fold change in MTMR2 levels in neurons nucleofected with control or *Rubicon* and *Mtmr2* shRNA plasmid (C) Fold change in Rubicon levels in neurons nucleofected with control or *Rubicon* and *Mtmr2* shRNA plasmid (A-C: N= 4 experiments, Mann-Whitney test). (D, E) Combined depletion of Rubicon and MTMR2 enhances basal autophagy of mitochondria in neurons. (D) Representative max projections of neurons nucleofected with Ctrl, *Mtmr2* and *Rubicon* shRNA and assayed for mitophagy using the protocol represented in Fig:7A. Outline of the neuronal soma for quantification is indicated using dashed lines in cyan. Yellow boxes indicate inset regions. (E) Number of mitophagolysosomes per soma (marked by colocalizing DMP Red and Lysotracker punctae) of neurons nucleofected with Ctrl, *Mtmr2* and *Rubicon* shRNA and assayed for mitophagy using the protocol represented in Fig:7A (N=4 experiments, two-tailed unpaired t-test, Scale bar = 5μm). (F, G) Knockdown of MTMR2 in WT cortical neurons to determine its role in regulating mitophagy. (F) Representative western blot from lysates of WT cortical neurons nucleofected with control or *Mtmr2* shRNA plasmids, and probed for MTMR2. (G) Fold change in MTMR2 levels in neurons nucleofected with control or *Mtmr2* shRNA plasmid. (N= 4 experiments, Mann-Whitney test).
Error bars indicate S.E.M.
