# Supplementary material for: Mitochondrial damage triggers the concerted degradation of negative regulators of neuronal autophagy
Source: Nat Commun. 2025 Aug 9;16:7367. doi: 10.1038/s41467-025-62379-5 (PMC12335601; doi:10.1038/s41467-025-62379-5)
Supplement: Supplementary file 2 — Reporting summary [file 41467_2025_62379_MOESM2_ESM.pdf]

nature portfolio

Corresponding author(s): Erika L. F. Holzbaur

Last updated by author(s): Jun 16, 2025

Reporting Summary

Nature Portfolio wishes to improve the reproducibility of the work that we publish. This form provides structure for consistency and transparency in reporting. For further information on Nature Portfolio policies, see our Editorial Policies and the Editorial Policy Checklist.

Statistics

For all statistical analyses, confirm that the following items are present in the figure legend, table legend, main text, or Methods section.

n/a	Confirmed	
☐	☒	The exact sample size (*n*) for each experimental group/condition, given as a discrete number and unit of measurement
☐	☒	A statement on whether measurements were taken from distinct samples or whether the same sample was measured repeatedly
☐	☒	The statistical test(s) used AND whether they are one- or two-sided<br>*Only common tests should be described solely by name; describe more complex techniques in the Methods section.*
☒	☐	A description of all covariates tested
☐	☒	A description of any assumptions or corrections, such as tests of normality and adjustment for multiple comparisons
☐	☒	A full description of the statistical parameters including central tendency (e.g. means) or other basic estimates (e.g. regression coefficient) AND variation (e.g. standard deviation) or associated estimates of uncertainty (e.g. confidence intervals)
☐	☒	For null hypothesis testing, the test statistic (e.g. *F*, *t*, *r*) with confidence intervals, effect sizes, degrees of freedom and *P* value noted<br>*Give P values as exact values whenever suitable.*
☒	☐	For Bayesian analysis, information on the choice of priors and Markov chain Monte Carlo settings
☒	☐	For hierarchical and complex designs, identification of the appropriate level for tests and full reporting of outcomes
☒	☐	Estimates of effect sizes (e.g. Cohen's *d*, Pearson's *r*), indicating how they were calculated

Our web collection on statistics for biologists contains articles on many of the points above.

Software and code

Policy information about availability of computer code

Data collection	All microscopic images were acquired on a PerkinElmer UltraView Vox spinning disk confocal on a Nikon Eclipse Ti Microscope with an Apochromat 100x 1.49 N.A. oil-immersion objective and a Hamamatsu CMOS ORCA Fusion (C11440-20UP) camera with VisiView (Visitron). Western blots were imaged in Odyssey CLx Infrared Imaging System
Data analysis	Volocity v6.3.1, Image Studio v5, Ilastik v1.3.0, GraphPad v10.1.1, FIJI/ImageJ v2.3.0/1.53f, Microsoft Excel (v16.81)

For manuscripts utilizing custom algorithms or software that are central to the research but not yet described in published literature, software must be made available to editors and reviewers. We strongly encourage code deposition in a community repository (e.g. GitHub). See the Nature Portfolio guidelines for submitting code & software for further information.

Data

Policy information about availability of data

All manuscripts must include a data availability statement. This statement should provide the following information, where applicable:

- Accession codes, unique identifiers, or web links for publicly available datasets
- A description of any restrictions on data availability
- For clinical datasets or third party data, please ensure that the statement adheres to our policy

Source data will be made publicly available on Nature Communications doi link for the manuscript, and on Zenodo.

Research involving human participants, their data, or biological material

Policy information about studies with human participants or human data. See also policy information about sex, gender (identity/presentation), and sexual orientation and race, ethnicity and racism.

Reporting on sex and gender	*Use the terms sex (biological attribute) and gender (shaped by social and cultural circumstances) carefully in order to avoid confusing both terms. Indicate if findings apply to only one sex or gender; describe whether sex and gender were considered in study design; whether sex and/or gender was determined based on self-reporting or assigned and methods used. Provide in the source data disaggregated sex and gender data, where this information has been collected, and if consent has been obtained for sharing of individual-level data; provide overall numbers in this Reporting Summary. Please state if this information has not been collected. Report sex- and gender-based analyses where performed, justify reasons for lack of sex- and gender-based analysis.*
Reporting on race, ethnicity, or other socially relevant groupings	*Please specify the socially constructed or socially relevant categorization variable(s) used in your manuscript and explain why they were used. Please note that such variables should not be used as proxies for other socially constructed/relevant variables (for example, race or ethnicity should not be used as a proxy for socioeconomic status). Provide clear definitions of the relevant terms used, how they were provided (by the participants/respondents, the researchers, or third parties), and the method(s) used to classify people into the different categories (e.g. self-report, census or administrative data, social media data, etc.) Please provide details about how you controlled for confounding variables in your analyses.*
Population characteristics	*Describe the covariate-relevant population characteristics of the human research participants (e.g. age, genotypic information, past and current diagnosis and treatment categories). If you filled out the behavioural & social sciences study design questions and have nothing to add here, write "See above."*
Recruitment	*Describe how participants were recruited. Outline any potential self-selection bias or other biases that may be present and how these are likely to impact results.*
Ethics oversight	*Identify the organization(s) that approved the study protocol.*

Note that full information on the approval of the study protocol must also be provided in the manuscript.

Field-specific reporting

Please select the one below that is the best fit for your research. If you are not sure, read the appropriate sections before making your selection.

☒ Life sciences ☐ Behavioural & social sciences ☐ Ecological, evolutionary & environmental sciences

For a reference copy of the document with all sections, see nature.com/documents/nr-reporting-summary-flat.pdf

Life sciences study design

All studies must disclose on these points even when the disclosure is negative.

Sample size	Sample size was not predetermined for any experiment. Sample size was chosen based on previously published literature and depending on experimental variability.
Data exclusions	Neurons which looked unhealthy under the microscope and showed signs of blebbing were excluded. Images of poor quality were not included during analysis.
Replication	For each experiment atleast three biological replicates from two independent sources have been used.
Randomization	Neurons isolated from each dissection were randomly assigned to the conditions of an experiment
Blinding	Blinding was not performed during data acquisition or analysis. All data for an experiment were acquired and analyzed under the same conditions.

Reporting for specific materials, systems and methods

We require information from authors about some types of materials, experimental systems and methods used in many studies. Here, indicate whether each material, system or method listed is relevant to your study. If you are not sure if a list item applies to your research, read the appropriate section before selecting a response.

Materials & experimental systems

n/a	Involved in the study
☐	☒ Antibodies
☐	☒ Eukaryotic cell lines
☒	☐ Palaeontology and archaeology
☐	☒ Animals and other organisms
☒	☐ Clinical data
☒	☐ Dual use research of concern
☒	☐ Plants

Methods

n/a	Involved in the study
☒	☐ ChIP-seq
☒	☐ Flow cytometry
☒	☐ MRI-based neuroimaging

Antibodies

Antibodies used

For western blotting and biochemical experiments: The following primary antibodies were used in this study: Rubicon at 1:1000 (CST #D9F7; RRID: AB_10891617), MTMR2 at 1:2000 (sc-365184; RRID: AB_10708283), MTMR5 at 1:500 (sc-393488; RRID: AB_3097714), Parkin at 1:1000 (CST #2132; RRID: AB_10693040), VPS34 at 1:2000 (NB110-87320SS; RRID: AB_1199455), Mitofusin-2 at 1:1000 (sc-100560; RRID: AB_2235195), ATG7 at 1:1000 (ab133528; RRID: AB_2532126), ATG5 at 1:1000 (ab108327; RRID: AB_2650499), GAPDH at 1:1000 (ab9484; RRID: AB_307274), Actin at 1:1000 (Sigma-Aldrich MAB1501R; RRID: AB_2223041), Tubulin at 1:2000 (CST #2148; RRID: AB_2288042), LC3B at 1:1000 (ab48394; RRID: AB_881433), Ubiquitin at 1:1000 (sc-8017; RRID: AB_628423), LAMP1 at 1:1000 (DSHB#1D4B, RRID: AB2134500), Mitofilin at 1:1000 (ab110329, RRID: AB10859613), HECTD1 at 1:2000 (ab101992, RRID: AB10711075), ATF4 at 1:1000 (CST 11815, RRID:AB_2616025), SCARB2 at 1:1000 (LSBio LS-B305, RRID:AB_2182974), Synapsin-1 at 1:2000 (Sigma AB1543P, RRID: RRID:AB_90757), RAB7 at 1:1000 (ab50533, RRID:AB_882241), BNIP3 at 1:500 (sc56167, RRID:AB_2066767), TBK-1 at 1:1000 (CST 3013, RRID:AB_2199749), COX2A at 1:2000 (ab198286, AB_2861364), GABARAP at 1:1000 (ab109364; RRID:AB_10861928), Cathepsin B at 1:1000 (CST31718, RRID:AB_2687580) ATP6V1E1 at 1:1000 (Sigma GW222284F, RRID: AB_1845192), MTM1 at 1:500 (Proteintech 13924-1-AP, RRID:AB_2147700), MTMR14 at 1:500 (Proteintech 14973-1-AP RRID:AB_2147828). The following secondary antibodies were used at 1:20,000 dilution: IRDye® 800CW Donkey anti-Mouse IgG (Li-cor #926-32212, RRID: AB_621847); IRDye® 680RD Donkey anti-Mouse IgG (Li-cor # 926-68072, RRID: AB_1095362); IRDye® 800CW Donkey anti-Rabbit IgG (Li-cor # 926-32213, RRID: AB_621848); IRDye® 680RD Donkey anti-Rabbit IgG (Li-cor # 926-68073, RRID: AB_10954442), IRDye® 680RD Donkey anti-Chicken Secondary Antibody (Li-cor #926-68075, RRID: RRID:AB_10974977).
For immunoprecipitation:Rubicon Ab (CST# E5J5V) , MTMR2 (sc-365184), Rabbit IgG (CST#2729S), Mouse IgG (Vector labs #I-2000-1)

For immunostaining: The following primary antibodies were used: p62 at 1:250 (ab56416, RRID: AB945626), LAMP1 at 1:100 (DSHB#1D4B, RRID: AB2134500), MAP2 at 1:500 (AB5622, RRID: AB91939). The following secondary antibodies were used at 1:1000 dilution- Alexa Fluor 488 Goat anti-Rat IgG (H+L) (A11006, RRID: AB2534074), Alexa Fluor 555 Goat anti-Rabbit IgG (H+L) (A21429, RRID: AB_2535850), Alexa Fluor 633 Goat anti-Mouse IgG (H+L) (A21052, RRID:AB_2535719).

Validation

Antibodies that either have been previously validated by their respective vendors or cited frequently in other research articles have been used. No antibodies were separately validated for this study.

Eukaryotic cell lines

Policy information about cell lines and Sex and Gender in Research

Cell line source(s)	HeLa-M cells source: a gift from Andrew Peden, Cambridge Institute for Medical Research, UK
Authentication	HeLa-M cells were authenticated by STR profiling
Mycoplasma contamination	Detection of mycoplasma in HeLa cells was done using MycoAlert detection kit (Lonza, LT07)
Commonly misidentified lines (See ICLAC register)	No misidentified lines have been used in this study

Animals and other research organisms

Policy information about studies involving animals; ARRIVE guidelines recommended for reporting animal research, and Sex and Gender in Research

Laboratory animals	Mus musculus: Strains used- C57BL/6J (RRID: IMSR_JAX:000664)] and Parkin-/- [B6.129S4-Prkntm1Shn/J (RRID: IMSR_JAX:006582)]
Wild animals	No wild animals were used in this study.
Reporting on sex	Sex of the mouse embryos or pups was not determined for the experiments. Cortical neurons from male and female embryos were pooled together post dissection. Similarly, astrocytes from either sex were pooled together.
Field-collected samples	No field-collected samples were used in this study.
Ethics oversight	All protocols for mouse work were approved by the Institutional Animal Care and Use Committee at the University of Pennsylvania

Note that full information on the approval of the study protocol must also be provided in the manuscript.

Plants

Seed stocks	*Report on the source of all seed stocks or other plant material used. If applicable, state the seed stock centre and catalogue number. If plant specimens were collected from the field, describe the collection location, date and sampling procedures.*
Novel plant genotypes	*Describe the methods by which all novel plant genotypes were produced. This includes those generated by transgenic approaches, gene editing, chemical/radiation-based mutagenesis and hybridization. For transgenic lines, describe the transformation method, the number of independent lines analyzed and the generation upon which experiments were performed. For gene-edited lines, describe the editor used, the endogenous sequence targeted for editing, the targeting guide RNA sequence (if applicable) and how the editor was applied.*
Authentication	*Describe any authentication procedures for each seed stock used or novel genotype generated. Describe any experiments used to assess the effect of a mutation and, where applicable, how potential secondary effects (e.g. second site T-DNA insertions, mosiacism, off-target gene editing) were examined.*
